# CCS52A2/FZR1, a cell cycle regulator, is an essential factor for shoot apical meristem maintenance in *Arabidopsis thaliana*

**DOI:** 10.1186/1471-2229-12-135

**Published:** 2012-08-08

**Authors:** Yajie Liu, Wei Ye, Beibei Li, Xiaojing Zhou, Yuhai Cui, Mark P Running, Kede Liu

**Affiliations:** 1 National Key Laboratory of Crop Genetic Improvement, Huazhong Agricultural University, Wuhan, Hubei, P. R. China; 2 Oil Crops Research Institute of the Chinese Academy of Agricultural Sciences, Key Laboratory of Biology and Genetic Improvement of Oil Crops, Ministry of Agriculture, Wuhan, P. R. China; 3 Agriculture and Agri-Food Canada, Southern Crop Protection and Food Research Centre, London, ON, N5V 4 T3, Canada; 4 University of Louisville, Louisville, KY, USA

## Abstract

**Background:**

Cell division and cell fate decisions regulate organ formation and function in plant growth and development. It is still unclear how specific meristematic regulatory networks operate with the cell cycle machinery to translate stem cell identity and maintenance into cellular behavior. In this study, we address these questions by analysis of a shoot apex defective mutant, namely *xcm9.*

**Results:**

Phenotypic analysis of the *xcm9* mutant reveals concomitant premature termination of floral shoots with frequent bifurcation of the shoot apices, stems, and flowers. Microscopic observations show irregular cell organization in shoot apical meristems of *xcm9*. Positional cloning revealed that *xcm9* is a loss of function allele of the *CCS52A2/FZR1* gene, which has previously been implicated in root development. Expression analysis demonstrated that *CCS52A2* maintains a higher transcriptional expression level in actively dividing tissue. Genetic studies indicated that the *CCS52A*2 gene functions together with *WUSCHEL* (*WUS*) and *CLAVATA3* (*CLV3*) in regulating the development of the shoot meristem, and also contributes to this regulation together with the chromatin remodeling pathway. In addition, fewer *xcm9* cells express *CYCLIN B1:1*, showing that cell cycle progression is disrupted in the mutant.

**Conclusion:**

We propose that the *CCS52A2* gene is a mediator that functions together with meristematic genes to regulate meristem organization, and cross-functions with chromatin regulators in cell cycle progression during shoot apical meristem development.

## Background

In higher plants, the vast majority of structures and organs, such as leaves, stems, roots, and flowers, are formed postembryonically from groups of undifferentiated cells, called meristems. In many species of plants, certain meristems are maintained throughout life. The activities of the root apical meristem (RAM) and the shoot apical meristem (SAM) determine root and shoot structure and function, respectively. Stem cells are confined to the centers of shoot and root apices, and their proliferation is maintained by signals that cells receive from the local environment [1]. They undergo precisely controlled division, which must be rapid enough to replenish cells lost to differentiation, but restricted enough to prevent overproliferation of undifferentiated cells. Adjacent to the stem cells, several cells in the SAM and RAM form the organizing center (OC) and quiescent center (QC), respectively. These cells coordinate with neighboring cells to establish the balance between proliferation and differentiation in the meristem niche [2].

Several meristematic genes form feedback networks that control this dynamic balance. In *Arabidopsis*, *WUS* encodes a transcription factor expressed in the OC, and its expression promotes the identity of distal meristem cells as stem cells, which themselves are characterized in part by *CLV3* expression [3,4]. *CLV3* encodes a peptide hormone expressed in the central apical surface of shoot and floral meristems and is necessary for controlling the size of the central zone (CZ) in SAMs [5,6]. *WUS* and *CLV3* form a feedback regulation loop: CLV3 acts as mobile intercellular signal to negatively regulate *WUS* transcription in the OC via the receptor proteins *CLV1*/*CLV2*/*CORYNE* (*CRN*), while *WUS* positively regulates *CLV3* expression [7-9]. Similar to the shoot meristem, the root QC maintains the stem state of the surrounding cells and prevents these cells from differentiating [10]. *WOX5* is the functional homolog of *WUS* expressed in the QC [11], and CLE40 is a CLV3-related peptide expressed in differentiated stele and columella cells [12,13]. Like the *CLV3-WUS* network in the shoot, *CLE40* and *WOX5* form a self-regulating network that controls the proliferation and differentiation of stem cells in the root [14].

Besides these vital meristematic regulatory genes, the organization and maintenance of cells in meristems are also modulated by several cell cycle control genes. It is still unclear whether the cell cycle machinery acts largely independently in regulating meristem organization, or acts by receiving the signals from meristematic pattern genes via unknown mechanisms. The cell division cycle protein CDC5, cyclin D3 (CYCD3), HBT/CDC27B, and the cyclin-dependent kinases A;1 (CDKA;1), CDKB2;1 and CDKB2;2 have been reported to be necessary for SAM and/or RAM development [15-19]. These proteins are among numerous cell cycle regulators, including other cyclins, CDKs, CDK inhibitors and CDCs, that precisely control the mitotic cell cycle during the four cell phases and several checkpoints to accomplish DNA replication and subsequent division. Once the need for the cell cycle regulators ends, they are degraded by ubiquitin-mediated proteolysis. Anaphase-Promoting Complex (APC), which functions as an E3 ubiquitin ligase that marks target cell cycle proteins for degradation by the 26 S proteasome, plays an important role in the phase transition of the cell cycle [20].

Some genes that are not considered to be part of the cell cycle regulator class of proteins also contribute to the development of meristems and are involved with the normal sequence of the cell cycle. Among these are the *MGOUN 1* (*MGO1*) and *FASCIATA 1* (*FAS1*) genes, which play critical roles in the fundamental organization and/or functioning of both the SAM and RAM [21]. *fas1* and *mgo1* are characterized by fasciated stems and short roots [22,23]. FAS1 is one subunit of *Arabidopsis* chromatin assembly factor-1 (CAF-1), which shows a conserved activity for chromatin assembly at the DNA replication fork in S phase. Loss of function of FAS1 alters the epigenetic marks at promoters of genes involved in activation of the G2 damage checkpoint, leading to inhibition of mitosis progression [24]. MGO1 is homologous to type IB topoisomerase, which has been reported to stabilize the epigenetic state of developmentally regulated genes and to affect gene expression in conjunction with the chromatin remodeling pathway in *Arabidopsis*[25]. An unknown mechanism is likely to be present that integrates the meristematic regulating network with the cell cycle machinery to translate SAM identity and maintenance into cellular behavior. The exact nature of this linkage and the mediator or mediators between these two networks is an important research question.

In this article, we report the isolation of a novel SAM defective mutant, *xcm9*, which displays clear signs of bifurcation and premature termination under our conditions. Positional cloning revealed that the *XCM9* gene encodes an activator of APC/C, namely CCS52A2. RT-PCR, promoter analysis and *in situ* hybridization assays show that the *CCS52A2* gene is broadly expressed in all organs tested, but has higher expression specifically in the shoot apices and root tips. Our genetic studies indicate that the *CCS52A2* gene functions together with *WUS* and *CLV3* in stem cell regulation, and also contributes to this regulation together with the chromatin remodeling pathway. Monitoring of *CYCB1:1* expression revealed that cell cycle progression is disturbed in *xcm9*. We propose that the *CCS52A2* gene is a mediator that regulates meristem organization, functions together with meristematic genes and cross-functions with chromatin regulators in cell cycle progression during SAM development.

## Results

### Mutations in the *XCM9* gene disrupt SAM development and maintenance

**Figure 1:**
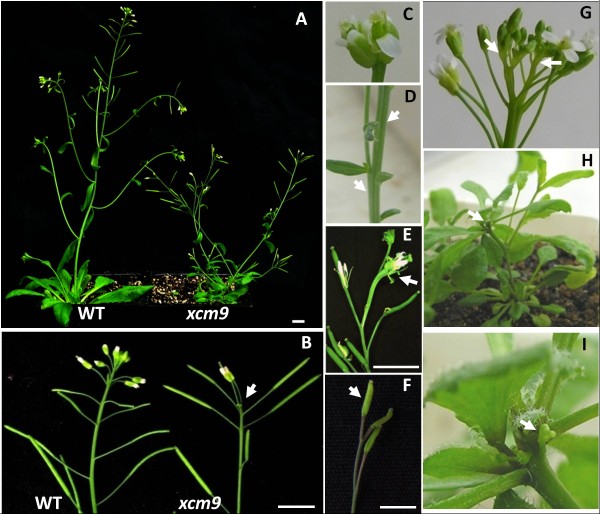
**Phenotypes of *xcm9*.** (**A**) and (**B**), The mature *xcm9* mutant displays premature termination of the inflorescence shoot in comparison with WT. Bars = 1 cm. (**C**), (**D**) and (**G**), Bifurcations in flowers (**C**), stems (**D**) and shoot apices (**G**). Bars = 1 cm. Arrowhead indicates the bifurcation. (**E**) and (**F**), Irregular terminal of Inflorescence shoot in *xcm9* displays as a fused flower (**E**) and a single silique (**F**). Bars = 1 cm. (**H**) and (**I**), Extreme premature termination of inflorescence shoot in *xcm9*. (**I**) close-up of (**H**). Arrowhead in (**I**) indicates flower buds arrested at an early developmental stage.

**Figure 2:**
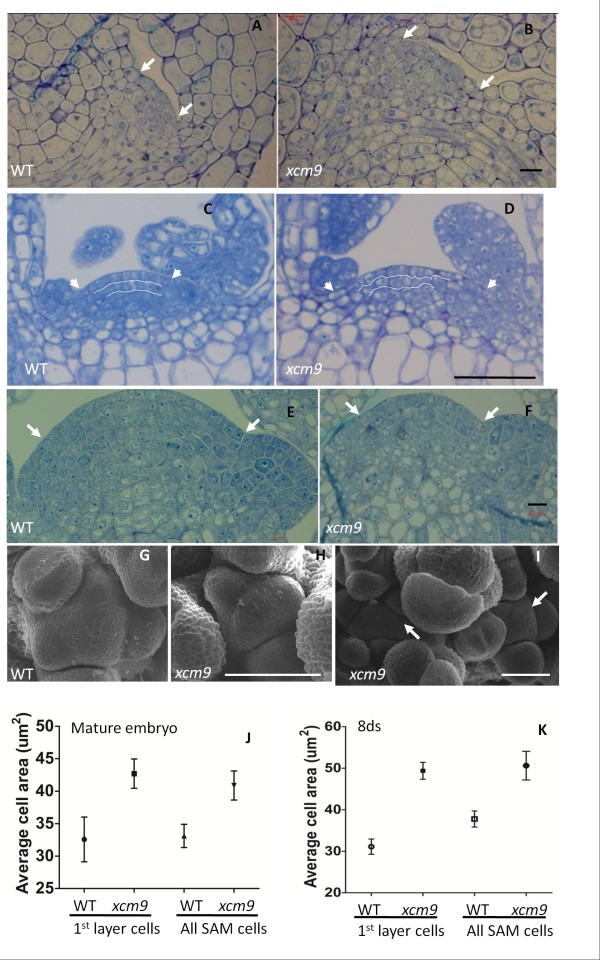
**Supplemental phenotypes of *xcm9*.** (**A**) to (**D**), Extreme premature termination of the inflorescence shoot in *xcm9*. (B**,D**) magnified image of (**A** and **C**) respectively. Arrowhead in (**B**) (**D**) indicates the tiny flower buds, arrested in the early development. (**E**) to (**G**), *xcm9* displays smaller organ sizes in stems, siliques and leaves compared with WT. Bars = 1 cm.

While screening for phenotypic mutants from MYB-family T-DNA insertion lines, we identified a shoot apex-defective mutant segregating in SALK_074403 from the SALK T-DNA collection [26]. This mutant was named *xcm9*. *xcm9* mutants demonstrate premature termination (Figure 1A, B) and bifurcations of shoot apices (Figure 1C, D, G). This premature termination is evident in the primary shoot and lateral inflorescences of *xcm9*, which have an average of 6.97 (range from 1–14, n = 39) flowers on main stems and 9.68 (range from 1–20, n = 39) flowers on lateral inflorescences, compared with an average of 39 (range from 28–46, n = 9) flowers on WT. In most cases, *xcm9* plants prematurely terminated with fewer flower buds and a senesced SAM (Figure 1B). But in some groups of shoot apices, the terminus had a flower stalk with a single flower or two fused flowers without an apparent SAM (Figure 1E, F), suggesting that the SAM is completely consumed by flower production at the terminus. Those single or several-fused flowers in the terminus always exhibited fewer flower organs (petals and sepals) and showed curved or fasciated stigmas (Figure 1E). In some extreme cases, the floral SAM did not generate any flowers but only terminated as a cluster of tiny buds, which were arrested during early stages of flower development (Figures 1H, I and 2A-D). Bifurcations were frequently observed in reproductive shoot apices (Figure 1G), flowers (Figure 1C) and stems (Figure 1C). Thus while only 12.8% of reproductive shoot apices of *ccs52a2-3* (n = 39) was observed to bifurcate, we can infer that more bifurcations occurred in the rosette during early vegetative development, since some *xcm9* mutants had more than one leaf initiation center. Subsequent results from local gene expression supported this (see below).

Besides the defective shoots, *xcm9* was reduced in height (Figure 1A), stem diameter (Figure 2E), silique length (Figure 2F) and leaf size (Figure 2G). The number of seeds per silique in *xcm9* was reduced, but the seed size was about 1/5 larger in both length (448.7 ± 23.8 μm for WT, 556.6 ± 42.8 μm for *xcm9*, p < 0.05) and in width (252.9 ± 26 μm for WT, 321.9 ± 24.1 μm for *xcm9*, p < 0.05).

### Cell organization of SAM is disordered in *xcm9*

**Figure 3:**
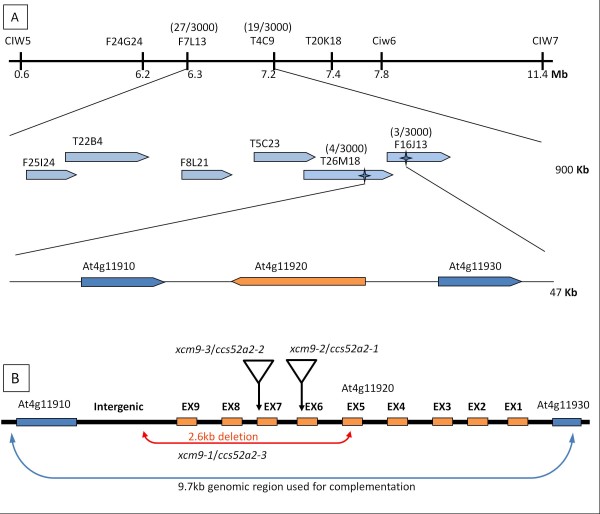
**The micro structures of SAMs in *xcm9*.** (**A**) and (**B**), SAM sections of mature embryos in WT (**A**) and *xcm9* (**B**). Bar = 10 μm. (**C**) and (**D**), SAM sections of 8-day-old seedlings in WT (**C**) and *xcm9* (**D**). Bar = 50 μm. (**E**) and (**F**), SAM sections of 28-day-old inflorecence shoots in WT (**E**) and *xcm9* (**F**). Bar = 10 μm. (**G**) to (**I**), Micro structures of inflorescence shoots of WT (**G**) and *xcm9* (**H**), (**I**) by scanning electron microscopy (SEM). Bar = 50 μm. The floral SAM the in primary shoot of *xcm9* (**H**) was smaller than that of WT (**G**). The floral SAM in lateral shoot of *xcm9* (**I**) shows bifurcation. Arrowhead indicates the two SAMs in one floral shoot. (**J**) and (**K**), The ranges of cell areas of 1st layer cells and all cells in SAMs of mature embryo (**J**) and 8-day-old plants (**K**).

To determine whether the defects of shoot apices in mutants were preceded by the abnormal development of the SAM, we visualized the cellular morphology of mutant meristems by semi-thin section. Cell organization was disrupted in the L1 and L2 layers of the SAM in 8-day-old *xcm9* plants compared with the highly organized cells in WT (Figure 3C, D). The width of SAMs in mature embryos and 8-day-old seedlings of *xcm9* were broadened to 41.3 ± 0.8 μm and 108.5 ± 5.8 μm, compared with 34.2 ± 0.6 μm and 75.2 ± 4.5 μm in the WT, respectively (Figure 3A-D). In addition, counting of cell numbers in mutant SAM semi-thin sections showed that there was no significant difference (student *t* test p = 0.15 > 0.05, n = 6) with the WT. Examination of the average cell areas in both the L1 layer and throughout the SAM both indicated that the enlargement of the SAM was mainly due to an increase of the meristem cell areas in the *xcm9* mutant in comparison with WT (Figure 3J, K). We also compared the floral SAMs between *xcm9* and WT. The flowering-stage SAMs from primary stems of *xcm9* were smaller (Figure 3F, H) than that of WT (Figure 3E, G), which is in contrast to SAMs in embryos and seedlings. Scanning electron microscopy (SEM) was used to investigate the overall structure of flowering-stage SAMs. The results indicate that the *xcm9* SAM in the primary floral shoot was smaller than WT, which is consistent with results from semi-thin sections; this may be due to premature termination of the floral shoot in *xcm9*. Bifurcated SAMs were usually observed in lateral floral shoots, and each of the splitting SAMs was surrounded by a ring of floral primordia (Figure 3I). All of the above suggests the size and cell organization of the SAM were both affected in conjunction with the phenotype of defective shoots in *xcm9*.

### A 2.6Kb deletion of *At4g11920* caused the shoot apex defect

To test if the mutant phenotype of *XCM9* gene cosegregated with the kanamycin resistance harbored in the T-DNA insert, we grew the mutant seeds on MS medium with kanamycin and found that they are kanamycin-sensitive, indicating that the kanamycin resistance gene may be silenced or lost. The T-DNA website (signal.salk.edu) [26] indicates that the T-DNA was inserted in the 3rd exon of the At1g18960 gene, which encodes a myb-like HTH transcriptional regulator family protein. Primers were designed from the flanking sequences of the T-DNA insertion to confirm its existence. Expected amplification was obtained, indicating that the kanamycin resistance gene was silenced in the mutant. Segregation analysis indicated that the T-DNA insertion did not cosegregate with the mutant phenotype, suggesting that the mutant phenotype is not caused by the T-DNA insertion.

**Figure 4:**
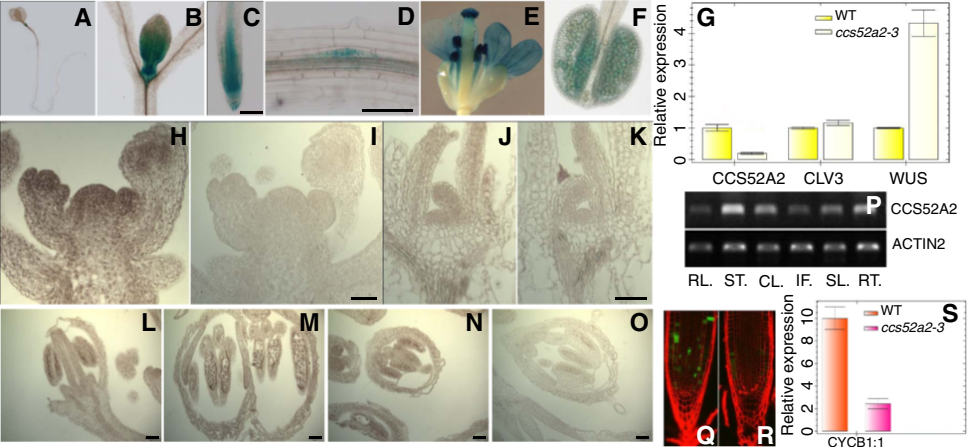
**Map based cloning of *CCS52A2*.** (**A**), Gene location of *CCS52A2*. The positions of mapping makers are shown. (**B**), The *CCS52A2* gene structure. The positions of the *ccs52a2-3* deletion mutation, *ccs52a2-1* (SALK_001978) and *ccs52a2-2* (SALK_073708C) insertion mutations, and 9.7 Kb genomic region used for complementation, are shown.

To clone the gene that was disrupted in the *xcm9* mutant, the *xcm9* mutant was crossed to ecotype Landsberg *erecta* (L*er*) for mapping. All F_1_ plants displayed a WT phenotype, whereas the F_2_ progeny segregated for *xcm9* and WT seedlings, indicating that the *xcm9* mutation is recessive in a single nuclear gene (*χ*^2^ = 0.06 < χ_0.05(1)_^2^ = 3.84). Bulked segregant analysis mapped the *xcm9* locus to the interval between CIW5 and CIW7 on chromosome 4. Further fine mapping located the *XCM9* locus between the two short sequence length polymorphism (SSLP) markers on bacterial artificial chromosomes (BACs) F7L13 and T4C9. Additional markers were used to fine-map the locus between SSLP markers on BAC clones T26M18 and F16J13 (Figure 4A). Sequencing of the genomic DNA amplified from this 47Kb region revealed a 2.6Kb deletion that starts from the 5th exon of At4g11920 and extends to the intergenic region between At4g11920 and At4g11910 in the *xcm9* mutant.

To verify whether the deletion in At4g11920 caused the shoot apex defects in *xcm9*, we next performed a complementation test by inserting a 9.7Kb genomic DNA fragment containing the 1.5Kb upstream sequence, the full-length coding region and 1.2Kb downstream sequences of At4g11920 (which includes the At4g11910 coding region) into heterozygous plants of *xcm9* (Figure 4B), because homozygous *xcm9* plants exhibit a strong phenotype and do not produce enough flowers for transformation. We found that all transgenic plants obtained in a homozygous *xcm9* background were phenotypically indistinguishable from WT plants, confirming that the apex defect phenotypes observed in *xcm9* were caused by the deletion of either At4g11910 or At4g11920. In addition, two SALK lines, SALK_001978 and SALK_073708C were identified with T-DNA insertions in exon 7 and 6 of At4g11920, respectively (Figure 4B). These two mutants showed similar phenotypes to *xcm9*, and were designated as *xcm9-2* and *xcm9-3*, respectively. These results together confirmed that the mutant phenotypes were caused by the mutation of At4g11920.

The At4g11920 gene consists of nine exons and eight introns (Figure 4B) and encodes a putative CDH1/CCS52A2/FZR1 protein (CDH1, also known as HCT1 for Homolog of CDC Twenty; CCS52A2, also known as a 52 kDa protein encoded by a Cell Cycle Switch gene), which contains WD40 repeats and is a component of APC/C, acting a co-activator and substrate recognizer [27,28]. Therefore, the *xcm9* mutant was renamed *ccs52a2-3*. *xcm9-2* corresponds to *ccs52a2-1,* and *xcm9-3* was renamed *ccs52a2-2*[29]. In *Arabidopsis*, *CCS52A2* was reported as stabilizing root meristem maintenance by acting in the distal region of the root and regulating the mitotic state of the QC [30]. Additionally, *CCS52A2,* under the regulation of the transcription factor EF1/DEL, promotes endoreduplication in cells of mature leaves [29]. In *Arabidopsis*, three *CDH1/FZR* homologs, *AtCCS52A1*, *AtCCS52A2* and *AtCCS52B*, have been previously identified [31]. All of the CCS52 (A1, A2 and B) proteins show a similar structure, with conserved C-box, CSM (Cdh1-specific motif), the IR motifs (APC binding domain) and the CBM motif (mitotic RVL cyclin binding motif) [31]. Importantly, *ccs52a2-3* lost the conserved potential CDK phosphorylation sites, IR and CBM motifs of CCS52A2, suggesting that this allele is a possible null mutant.

### *CCS52A2* mRNA is broadly expressed

**Figure 5:**
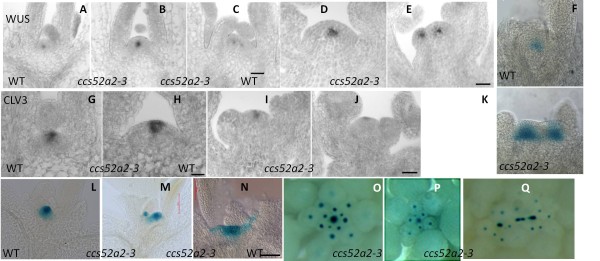
**Expression pattern of CCS52A2 in *Arabidopsis*.** (**A**) to (**F**), CCS52A2p::CDS::GUS expression in shoot meristems (**A**) (**B**), root tips (**C**), lateral root (**D**), petals (**E**) and mature pollen (**F**) in 8-day-old seedlings. Bar = 100 μm. (**G**), mRNA expression results of *CCS52A2*, *CLV3* and *WUS* in SAM of 10-day-old seedling (WT and *ccs52a2-3*) from real time-PCR. (H) to (O), CCS52A2 expressions in SAMs of inflorescence shoot (**H**) and 8-day-old seedling (**J**) and pollen (**L**) (**M**) (**N**) by using *in situ* hybridization. (**I**) (**K**) (**O**) Hybridization with sense probes as a control. Bar = 50 μm. (**P**), CCS52A2 expression in various *Arabidopsis* tissues by RT-PCR. *ACTIN2* is shown as a loading control. RT = Rossette leaves, ST = stem, CL = Cauline leaves, IF = Inflorescence, SL = Silique, RT = Root. (**Q**) and (**R**), CYCB1;1::GFP expression in roots of 8-day-old seedlings of WT (**Q**) and *ccs52a2-3* (**R**). (**S**), mRNA expression results of *CYCB1;1* in SAM of 10-day-old seedling (WT and *ccs52a2-3*) from real time-PCR.

RT-PCR analysis indicated that CCS52A2 had a broad expression profile including roots, rosette leaves, stem, cauline leaves, inflorescences and siliques (Figure 5P). The expression of *CCS52A2* was also examined in 8-day-old plants by a histochemical GUS assays, using transgenic plants expressing the CCS52A2::GUS fusion protein under the control of the CCS52A2 native promoter. Strong expression was observed in shoot apices and root tips (Figure 5A-D). To determine the expression region of *CCS52A2* mRNA, *in situ* hybridization was carried out. The results were consistent with that of the GUS assay, presenting strong signals in the entire SAM (Figure 5H, J), lateral leaf primordia in 8-day-old seedlings (Figure 5J), flower primordia (Figure 5H), and pollen (Figure 5E, F, L-N) of different stages in 28-day-old plants.

### The expression patterns of *WUS* and *CLV3* were altered in the *ccs52a2-3* mutant

The disordered organization of SAM in *ccs52a2-3* and high expression of CCS52A2 in the shoot apex prompted us to investigate whether the expression patterns of two essential meristematic genes, *WUS* and *CLV3*, are affected in the mutant. qRT-PCR results using dissected SAM from 10-day-old seedlings indicated that *WUS* was expressed at a higher level in *ccs52a2-3*, while *CLV3* maintained slightly higher mRNA expression levels in *ccs52a2-3* mutants when compared with WT (Figure 5G).

**Figure 6:**
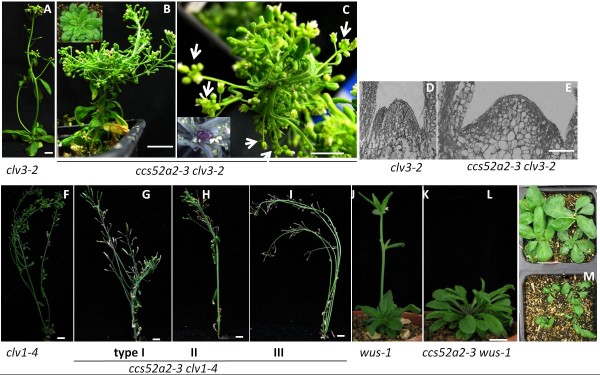
***WUS *and *CLV3 *expression in WT and *ccs52a2-3.*** (**A**) to (**C**), *WUS* expression in the SAM of 8-day-old seedling of WT (**A**) and *ccs52a2-3* (**B**) and (**C**). Bar = 50 μm. (**D**) and (**E**), *WUS* expression in the SAM of 30-day-old inflorescence shoots of WT (**D**) and *ccs52a2-3* (**E**). Bar = 50 μm. (**F**) and (**K**), pWUS::GUS expression in the SAM of 8-day-old WT (**F**) and *ccs52A2-3* (**K**). Two *WUS* expression domains are visible in *ccs52a2-3* (**K**). (**G**) and (**H**), *CLV3* expression in the SAM of 8-day-old seedling of WT (**G**) and *ccs52a2-3*(**H**). Bar = 50 μm. (**I**) and (**J**), *CLV3* expression in the SAM of 30-day-old inflorescence shoots of WT (**I**) and *ccs52a2-3* (**J**). Bar = 50 μm. (**L**) to (**N**), pCLV3::GUS expression in 8-day-old seedling SAM of wild-type (**L**) and *ccs52a2-3* (**M**) (**N**). Bar = 50 μm. (**O**) to (**Q**), pCLV3::GUS expression in the floral shoot SAM of WT (**O**) and *ccs52a2-3* (**P**)(**Q**).

To further examine the expression patterns of these meristematic genes in the SAM, mRNA *in situ* hybridization assay of *WUS* and *CLV3* genes were carried out. The expression of *WUS* in WT is restricted in a small region composed of a few cells in the center of the SAM (Figure 6A, D) [4]. However, in the SAM of 8-day-old *ccs52a2-3* seedlings, *WUS* expressed in one enlarged domain at higher levels (Figure 6B), or in two distinct nearby domains (Figure 6C), suggesting that enlarged or multiple OC zones are formed, which is consistent with the broadening SAMs and bifurcations of the stem. In contrast to seedlings, the reproductive SAMs of the mutant had either no detectable or decreased expression of *WUS* (Figure 6E) in comparison with WT (Figure 6D), which suggests that the size reduction and premature termination of the floral shoot meristems in *ccs52a2-3* mutants may be caused by a loss of *WUS* activity.

In WT, *CLV3* mRNA accumulates in a small zone of cells in the first three layers at the meristem apex (Figure 6G, I) [6]. Compared with WT, the SAMs of 8-day-old *ccs52a2-3* seedlings that were examined exhibited increased *CLV3* expression (Figure 6H). However, most of the 28-day-old *ccs52a2-3* plants (n = 10) had a slightly wider but shallower expression of *CLV3* in the reproductive SAM sections compared to limited expression in the central surface of WT SAM (Figure 6J), while some didn’t show a significant difference with WT.

To verify the results from *in situ* hybridization, we monitored the expression of *WUS* and *CLV3* using GUS reporters (p*WUS*::GUS and p*CLV3*::GUS) in WT and *ccs52a2-3*. The p*WUS*::GUS assay presented a similar expression pattern as RNA *in situs*: enlarged and sometimes dual expression domains in the *ccs52a2-3* background (Figure 6K). In addition, the p*CLV3*::GUS location in *ccs52a2-3* SAM exhibited two nearby domains of *CLV3* expression in 6 of 22 8-day–old seedlings examined (Figure 6M), which indicated that two separate *WUS*-*CLV3* supporting systems of meristem organization had been established as early as the 8-day-old stage. It was also discovered that *CLV3* showed broad expression in some first layer cells that flank the major expression zone in 7 of 22 SAMs of *ccs52a2-3* seedlings (Figure 6N), while the remaining 9 of 22 *ccs52a2-3* SAMs exhibited a single *CLV3* expression region. Additionally, the floral SAMs in main stems of *ccs52a2-3* always displayed decreased *CLV3* expression (Figure 6P), but in some lateral branches multiple CLV3 expression regions were observed (Figure 6Q). These were consistent with the observations of multiple SAMs in lateral branches (Figures 1G, 3I) and smaller SAMs in main stems (Figure 3F, H).

### *ccs52a2-3* is synergistic to *wus* and *clv* mutants

**Figure 7:**
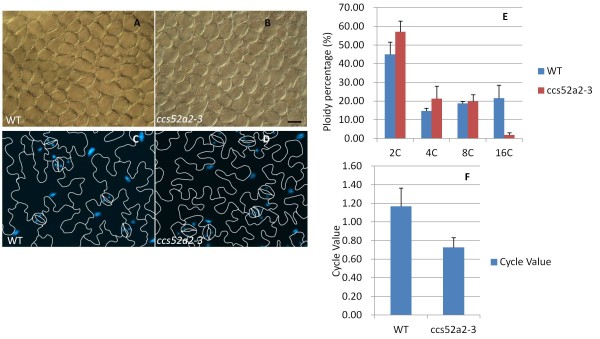
**Morphlogy of *ccs52a2-3 clv3-2*, *ccs52a2-3 clv1-4 *and *ccs52a2-3 wus-1 *double mutants.** (**A**) to (**G**), *clv3-2*(A) and *ccs52a2-3 clv3-2*(B) (**C**)*.* Arrowheads indicate shoot apices from one shoot of *ccs52a2-3 clv3-2* (C). *ccs52a2-3 clv3-2* has enlarged SAM (E) (from section) of 8-day-old seedlings compared with single mutant *clv3-2* (**D**). Bars = 1 cm in (**A**)-(**C**); Bar = 50 μm in (**D**), (**E**). (**F**), Mature *clv1-4* plant. Bars = 1 cm. (**G**) to (**I**), Mature *ccs52a2-3 clv1-4* double mutant. Type I (**G**), type II (**H**) and type III (**I**). Bars = 1 cm. (**J**) and (**K**), 42-day-old *wus-1* single mutant (**J**) and *ccs52a2-3 wus-1* double mutant (**K**). Bars = 1 cm. (**L**) and (**M**), Two weeks old *wus-1* (**L**) and *ccs52a2-3 wus-1* (**M**).

To test if genetic interactions exist between *CCS52A2* and the *WUS*, *CLV1* and *CLV3* genes, we created the double mutants *ccs52a2-3 wus-1*, *ccs52a2-3 clv1-1*, *ccs52a2-3 clv1-4* and *ccs52a2-3 clv3-2* by crossing *ccs52a2-3* with *wus-1*, *clv1-1*, *clv1-4* and *clv3-2*, respectively. *clv1-4* and *clv3-2* produce enlarged shoot and floral meristems relative to WT and are considered the strongest alleles of *clv1* and *clv3* mutants (Figure 7A, F) [5,32]. Double mutants with *clv3-2* had a novel phenotype: *ccs52a2-3 clv3-2* plants (n = 48) consistently had many more rosette leaves with larger shoot meristems (Figure 7E) and exhibited a delayed transition from vegetative phase to reproductive phase compared to WT and single mutants. Additionally, *ccs52a2-3 clv3-2* double mutants displayed severe fasciation in reproductive SAMs and stems (Figure 7B, C). More severe bifurcations (7 shoot apices on average and sometimes more than 30 in a single stem, depending on nutritional conditions) were present in *ccs52a2-3 clv3-2* double mutants compared to *ccs52a2-3* single mutants, which generally produced 2 or 3 shoot apices from bifurcations. We were unable to count the exact number of shoot apices resulting from such extreme bifurcations because of the twisted stems.

CLV1 is the direct downstream target of CLV3 in the CLV3-WUS pathway [9], so *ccs52a2-3 clv1* was expected to have similar phenotype to *ccs52a2-3 clv3*. Observations of *ccs52a2-3 clv1-1* and *ccs52a2-3 clv1-4* double mutants showed similar fasciation in shoot apices as in the *ccs52a2-3 clv3-2* double mutants. The *ccs52a2-3 clv1-4* (n = 39) displayed bifurcation at a very high frequency, over 50% under our conditions, compared to 12.8% for *ccs52a2-3* (n = 39). All *ccs52a2-3 clv1-4* double mutants were classified into three types according to the width of fasciated stems (Figure 7G-I). Notably, type I had the widest major stem, followed by type II and then the type III. The type I and type II double mutants had no lateral branches originating from the rosette leaves, but type III did. Most of the *ccs52a2-3 clv1-4* double mutants prematurely terminated in the formation of several flowers in lateral branches, which signifies that *clv1-4* can’t rescue premature termination in *ccs52a2-3* by enlarging SAM size in the absence of CLV function. Furthermore, flowers in both *ccs52a2-3 clv3-2* and *ccs52a2-3 clv1-4/clv1-1* double mutants appeared to be typical of *clv* flowers, with increased floral organ number, including 4–8 carpels and 5 petals. The severe SAM bifurcations and fasciation in *ccs52a2-3* clv3*-2* and *ccs52a2-3 clv1-4* double mutants revealed a synergistic interaction between CCS52A2 and the CLV pathway, with CCS52A2 being involved in independent pathways but in the same general process of SAM development and maintenance as CLV3 and CLV1.

*WUS* is another important gene in the organization and identity of SAM cells, as *WUS* creates a feedback regulation loop with *CLV1* and *CLV3*[7]. The primary shoot meristem of *wus-1* seedlings fails to maintain itself, and cells of the meristem differentiate, halting further shoot growth until adventitious meristems form to initiate further leaves and shoots [3,4]. To test the interaction of *WUS* with *CCS52A2*, we constructed the *ccs52a2-3 wus-1* double mutant. 34% of 12-day-old progeny of *ccs52a2-3/+ wus-1/+* showed a defective SAM that resembled the *wus-1* single mutant, which was higher than the 24% of *wus-1/+* self progeny (Table 1). Thus *ccs52a2-3 wus-1* double mutant seedlings (Figure 7M) appeared phenotypically identical to *wus-1* single mutants, displaying serious defects in SAM organization, including the inability to establish normal shoot meristems followed by the development of secondary adventitious-like meristems, which resulted in more rosette leaves (Figure 7K). But the SAM defects in most older *ccs52a2-3 wus-1* double mutants were enhanced, producing more rosette leaves than those in *wus-1* single mutants and delaying transition from the vegetative apex into the reproductive apex as is seen in *wus-1*. 100% of the 42-day-old *ccs52a2-3 wus-1* plants (n = 30) did not develop determinate inflorescence shoots, compared with 75% of *wus-1* single mutants (n = 42) harboring flowering shoots at the same age under our conditions (Table 1).

**Table 1:** **Shoot Meristem Defects in *wus ccs52a2-3 *Plants**

**Seedling meristem**			**Inflorescence shoots**			
**Genotype of parent plants**	**n**	**Arrested (%)**	**Genotype**	**42-day-old**		
				**n**	**Indet.(%)**	**Det.(%)**
wus-1−/+	103	24	wus-1	47	25	75
ccs52a2-3	37	0	ccs52a2-3	34	0	100
ccs52a2-3 wus-1−/+	105	34	ccs52a2-3 wus-1	30	100	0

Primary seedling shoot meristem was analyzed in 12-day-old plants. det., determinate shoot. Indet., indeteminated shoot.

### *CCS52A2* controls cell size and ploidy in differentiated cells during cell division

**Figure 8:**
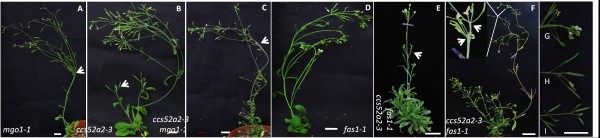
**Cell size and ploidy change in the mature leaves of *ccs52a2-3 *plants*.*** (**A**) and (**B**), Comparison of cell size of mesophyll cells in 5^th^ mature leaves of 3 week old WT (**A**) and *ccs52a2-3* (**B**) plants. Bar = 50 μm. (**C**) and (**D**), Comparison of cell size of abaxial epidermal cells in 5^th^ mature leaves of 3 week old WT (**C**) and *ccs52a2-3* (**D**) plants. (**E**), Ploidy distribution was determined by isolating nuclei from abaxial epidermal cells in 5^th^ mature leaves (n = 5). Mean values ± SD are shown. (**F**), The comparison of Cycle Value of 5^th^ mature leaves in WT and *ccs52a2-3* (n = 5). Mean values ± SD are shown.

In a previous study, CCS52A2 was found to control the endocycle, as indicated by the low Endoreduplication Index (EI) of mature leaves of the knockout lines CCS52A2^KO^ (analysis with *ccs52a2-1* mutant) and the increased number of cells with a high DNA ploidy level in CCS52A2^OE^ leaves [29]. *ccs52a2-3* mutants appeared dwarfed and had smaller leaves, stems and siliques in comparison with WT (Figures 1A and 2E-G). We hypothesized that these phenotypes might be due to decreased cell quantities and/or smaller cell sizes, so we analyzed the cell size and number of mature 5^th^ leaves. In comparison to WT, the 5^th^ mature leaves of *ccs52a2-3* mutants show a decrease of 13.58% in mesophyll cell size (Figure 8B) and a decrease of 70.61% in total mesophyll cell number (13039.6 ± 2964.3 in *ccs52a2-3*; 44361.8 ± 6767.2 in WT, Student *t* test p < 0.05), and a significant cell size decrease (2226.4 ± 61.6um^2^ in *ccs52a2-3*; 2576.2 ± 113.7um^2^ in WT, p < 0.05) was also detected in abaxial epidermal cells (Figure 8D). To test if the cell size decrease corresponds with lower ploidy levels, we measured the nuclear size of the abaxial epidermal cells in leaf peels. Statistical analysis demonstrated that that the mutant leaf cells with 16C ploidy made up a significantly lower percentage than in WT, while the mutant leaf cells of 2C ploidy showed a slightly higher percentage in the mutant (Figure 8E). The Cycle Value (CV) of 5^th^ leaves in the *cca52a2-3* plant was lower (p < 0.05) than the WT (Figure 8F) [33], consistent with lower EI of CCS52A2^KO^ leaves determined by flow cytometry in the previous report [29]. Therefore, we believed that loss of CCS52A2 function results in the decrease in cell size and ploidy level in mature leaves. However, as measured by flow cytometry, the CV of SAM cells in 8-day-old *ccs52a2-3* seedlings was unaffected in comparison with WT (data not shown). Despite the enlarged cell area of meristem cells in mutant SAMs (Figure 3J, K), the cell ploidy was not correspondingly changed, which means, unlike in the leaf, *CCS52A2* did not induce the onset of endocycle in the shoot meristems. The earlier published data of the RAM in *CCS52A2* knockout lines (*ccs52a2-1, ccs52a2-2*) also showed that CCS52A2-mediated control over root development did not involve endoreduplication [30].

CYCB1;1 was revealed as one of the possible substrates of APC/C to interact with CCS52s (A1, A2 and B) in *Arabidopsis*[31]. Since *CCS52A2* acts at G2/M to control mitosis exit and endocycle entry, we checked the expression of the G2/M transition reporter gene CYCB 1;1 in *ccs52a2-3* mutants. We monitored GFP reporters in the root tips of marker lines harboring CYCB1;1::GFP in a *ccs52a2-3* mutant background by confocal microscopy. The results showed that the number of RAM cells expressing CYCB1;1 was dramatically reduced, and the range of cells expressing CYCB1;1 in root was shorter in 7-day-old *ccs52a2-3* compared with WT (Figure 5R). In addition, qRT PCR was carried out to quantify the expression levels of CYCB1;1 in the shoot. The seedling SAM of *ccs52a2-3* showed lower expression level in comparison with that of WT (Figure 5S). Reduction of CYCB1;1 expression in both the SAM and RAM indicated that the cell cycle process was disturbed in *ccs52a2-3* mutants.

### *CCS52A2* interacted with *MGO1* and *FAS1*

**Figure 9:**
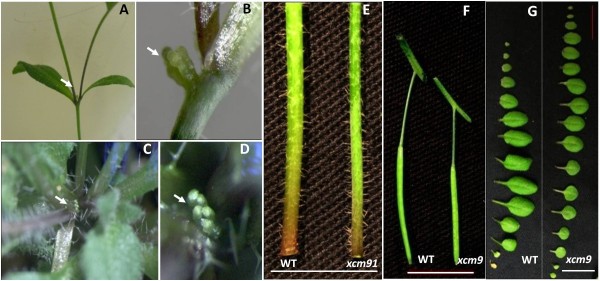
**Morphology of mature *mgo1-1*, *ccs52a2-3*, *ccs52a2-3 mgo1-1*, *fas1-1*, and *ccs52a2-3 fas1-1*.** (**A**), *mgo1-1*. The arrowhead indicates the bifurcations in stem. (**B**), *ccs52a2-3*.The arrowhead indicates premature termination in the primary shoot. (**C**), *ccs52a2-3 mgo1-1* double mutant. The arrowhead indicates similar bifurcations in the stem as the *mgo1-1* single mutant. (**D**), *fas1-1.* (E) and (F), *ccs52a2-3 fas1-1* double mutant, demonstrating bushy leaves (**E**) and enhanced bifurcation (**F**). Arrowheads indicated disturbed flower phyllotaxy (**E**) and two stems of bifurcation (**F**). (**G**) to (**I**), Floral shoot apex of *fas1-1*(G), *ccs52a2-3* (**H**) and *ccs52a2-3 fas1-1* (**I**). The *ccs52a2-3 fas1-1* showed premature termination in floral shoot in comparison with *fas1-1* single mutant at the same age. All bars = 1 cm.

The *mgo1-1* and *fas1-1* mutants display fasciation and bifurcation in both vegetative and reproductive development [22,23], similar to the phenotype of *ccs52a2-3* mutants. The similarity of the phenotypes compelled us to figure out whether these genes act in the same pathway and/or affect the same downstream processes as *CCS52A2*. To study their genetic relationship we constructed *ccs52a2-3 fas1-1* and *ccs52a2-3 mgo1-1* double mutants. The *ccs52a2-3 mgo1-1* double mutants were indistinguishable from *mgo-1* (Figure 9A, C) under our conditions, suggesting that *MGO1* may be epistatic to *CCS52A2*. The *ccs52a2-3 fas1-1* double mutants possessed many characteristics of both parents, but can be easily distinguished from either single mutant (Figure 9D-I). They have narrow leaves and disrupted floral phyllotaxy as in *fas1-1*, with bifurcation and premature termination characteristic of *ccs52a2-3*. However, the bifurcation ratio of inflorescences is higher than *ccs52a2-3* single mutants (71% of *ccs52a2-3 fas1-1*, n = 32; 12.8% of *ccs52a2-3*, n = 39). Many *ccs52a2-3 fas1-1* double mutants displayed a large number of rosette leaves with a bushy appearance in the mature plant (Figure 9E). The novel phenotype of *ccs52a2-3 fas1-1* double mutants revealed that FAS1 and CCS52A2 are involved in the same downstream process.

## Discussion

### *CCS52A2* is necessary for the normal structure and function of the SAM in *Arabidopsis*

This article demonstrates that loss of CCS52A2 function in *ccs52a2-3* mutants led to novel morphological defects in the SAM. The *ccs52a2-3* mutant displays concomitant premature termination of floral shoots with frequent bifurcation of the shoot apices, stems, and flowers. These defects in meristems indicate that the *CCS52A2* gene is essential for the proper maintenance and function of the SAM in plant development. Interestingly, the vegetative meristem and inflorescence meristem were larger and smaller than that of WT, respectively. One possible explanation for this is that that the double WUS expression zones in the 8-day-old mutant are indicative of bifurcation originating at this time. Lateral splitting results two smaller SAMs, each with a reduced WUS expression domain, and which later give rise to smaller inflorescence SAMs that terminate prematurely.

Expression changes of the major SAM regulator genes *WUS* and *CLV3 in ccs52a2-3* were observed. Ectopic *CLV3* expression was detected in the first layer of the SAM (Figure 6N), and the presence of more than one *WUS* and/or *CLV3* expression region in a single SAM of *ccs52a2-3* seedlings reveals a rare maintenance pattern in higher plant meristems. All of these abnormal expression patterns of SAM regulators indicate that malfunction of CCS52A2 affects cell identity in meristems. Previously published data show that the CCS52A2 gene plays a role in QC identity in root development, as the RAM organizing genes show irregular expression domains and locations and a failure to maintain the low mitotic activities of cells in QC zone in *ccs52a2-1* root [30]. We here provide evidence that the expression of SAM organizers are also altered in *ccs52a2-3*, and duplicate individual CLV3-WUS regulatory centers are sometimes generated in the mutant SAM. One possible explanation for this is that the proliferation of cells in OC zone results from a partial loss of cell division control, leading to formation of new and separate CLV3-WUS regulation systems in a single SAM of the mutant. Considering the role of CCS52A2 in the root, we give a hypothesis is that the duplicated OCs may somehow result from the failure to maintain low mitotic levels in certain SAM cells, with more cells available to fulfill certain roles.

### *CCS52A2* synergistically interacts with SAM organizers

We suspect that *CCS52A2* regulates the development of meristems by indirectly interacting with the *WUS* and *CLV3* genes. This hypothesis is firstly supported by up-regulated expression of *CLV3* and *WUS* in the SAM of *ccs52a2-3* seedlings (Figure 5G). Additionally, the more severe phenotypes seen in *ccs52a2-3 clv3-2* and *ccs52a2-3 clv1-4* double mutants indicate that *CCS52A2* may function in separate genetic pathways but are involved in the same process with *CLV1* and *CLV3*, and disrupting multiple modes of cell division regulation in meristems leads to a greater likelihood of a very large loss of cell division control and massive overproliferation. The finding of more severe bifurcations from the larger SAMs (compared with any single mutants) of *ccs52a2-3 clv* double mutants reveals that the bigger the size of the SAM, the more bifurcations occurred. *clv* single mutant exhibits an expanded SAM but has not been reported to undergo bifurcation.

The enhanced *wus-1* phenotype in *ccs52a2-3 wus-1* double mutant indicated that *CCS52A2* contributes to meristem establishment and leaf initiation together with *WUS*; however, *CCS52A2* also acts in an independent pathway as well.

### CCS52A2 functions in the SAM and stimulates the onset of endocycle in mature leaves

Previous studies suggest that CCS52A2 is an activator of APC/C, which mediates protein proteolysis during mitosis [30,31,34]. Therefore, it could regulate SAM maintenance and development by regulating cell division. Our hypothesis is consistent with cell cycle impairment, as indicated by the lower CYCB1;1 expression in RAM and SAM of *ccs52a2-3* (Figure 5R). However, the enlargement of the SAM is mainly due to enlarged cell areas and not increased cell number, which indicates that cell proliferation is still under control in mutant SAMs even if impairment exists in the cell cycle. *CCS52A2* has also been reported to promote the exit from the cell cycle into the endocycle, and leads to endoreduplication by association with APC/C in *Medicago truncatula*[28,34]. In *Arabidopsis*, similar effects are seen in CCS52A2 knockout (KO) and overexpression (OE) lines [29]. However, in this paper, the unchanged CV (corresponding to ploidy level) of the SAM indicates that the enlarged stem cells are not due to the entrance into endocycle in *ccs52a2-3.* This is consistent with previous studies that *CCS52A2* is not correlated with the endocycle in the RAM in CCS52A2^KO^, which exhibits a normal Endoreduplication Index in the mutant [30]. But in mature leaves, the presence of smaller cells with lower CV of cells in *ccs52a2* mutants indicates a role for CCS52A2 in these tissues. Taken together, these studies further supports the idea that *CCS52A2* only has obvious effects on modulating endocycle onset in differentiating cells and not in undifferentiated cells.

*CCS52A1*, a homolog of *CCS52A2*, has also been reported to control the endocycle of cells in mature leaves [35]. The similar ploidy changes in null mutants of *CCS52A1* and *CCS52A2* signify these two genes overlap in function in endoredupliction onset, but that they are not redundant.

### *CCS52A2* was involved in chromatin remodeling

Plants carrying a mutation in the MGO1 gene exhibit some phenotypes similar to *ccs52a2-3* mutants [22], such as bifurcation of stems. *MGO1* encodes an *Arabidopsis* Type IB DNA Topoisomerase, which functions in the relaxation of supercoiled DNA and acts in a number of different DNA metabolic processes, including replication, transcription, repair, and chromatin compaction [36,37]. It has been reported that MGO1 cooperates with chromatin regulators and is necessary for the maintenance of several epigenetically regulated genes [25]. The indistinguishable phenotypes of *ccs52a2-3 mgo1-1* double mutants compared to the *mgo1-1* single mutant revealed that these two genes might be involved in the same pathway. Recently, it was found that mutation of *MGO1* suppresses ectopic *WUS* activity and enhances stem cell defects in *wus* mutants [25]. Likewise, lack of *CCS52A2* enhances the SAM defect in *wus-1,* which further supports a functional relationship between *MGO1* and *CCS52A2*, and imply that their functions may converge to a common downstream process with *WUS* in stem cell regulation [25]. MGO1 and CCS52A2 proteins are both factors regulating cell cycle processes. It would be interesting to further investigate the molecular nature of the link between CCS52A2 and MGO1.

*fas1* is another fasciated mutant that displays stem bifurcation, as do *ccs52a2-3* and *mgo1*. *FAS1* encodes one subunit of the chromatin assembly factor (CAF-1), which coordinates nucleosome assembly during DNA replication and repair by depositing newly synthesized histone H3 and H4 [38]. Defects in *FAS1* lead to a halt in the progression through G2 by up-regulating several genes involved in the G2 DNA checkpoint, which it accomplishes by changing epigenetic marks of their promoters [24]. The novel phenotypes of *ccs52a2-3 fas1-1* double mutants demonstrate the two genes have a synergistic interaction in SAM development. The shared phenotypes of plants that have defects in either *FAS1*, *MGO1* or *CCS52A2* reveal a possibility that these three genes might be involved in the same downstream processes. Thus, FAS1 modulates the epigenetic states of genes involved in the G2 damage checkpoint, while MGO1 acts in DNA repair and maintains the expression of several epigenetically regulated genes [24,25]. Additionally, studies on nodule organogenesis indicate that CCS52A proteins mediate the degradation of mitotic cyclins in the proper phases of the cell cycle to inactivate CDKs, eventually blocking the G2 to M transition and thereby triggering endoreduplication [39]. Previous expression profile analysis of different cell samples in the SAM reveals an enrichment of DNA repair and chromatin modification pathways in stem cells, which suggests that the maintenance of flexible chromatin may facilitate the dynamic balance of gene expression during SAM development [40]. These results lend further evidence to the hypothesis that CCS52A2 might have a function related to triggering the G2/M transition, and that the signal pathways of CCS52A2, FAS1, and MGO1 are all involved in epigenetic maintenance and the activation of the G2 checkpoint.

## Conclusions

In this study, we showed that mutations in the *CCS52A2* gene disrupted the normal structure and function of the SAM in *Arabidopsis*. *CCS52A2* modulates the expression domains of the meristem regulatory gene *WUS* and *CLV3*. Moreover, double mutant analyses illustrates that *CCS52A2* synergistically interacts with SAM organizers. The cell cycle was disturbed in the absence of *CCS52A2*, as indicated by reduced expression of CYCB1; 1. *CCS52A2* is involved in control of cell size and ploidy in differentiated cells during cell division. Furthermore, *CCS52A2* is involved in chromatin remodeling with *FAS*1 and *MGO1*, which were previously reported to be involved in the activation of the G2 checkpoint and epigenetic maintenance. We propose that *CCS52A2* regulates meristem organization, functions together with meristematic genes and cross-functions with chromatin regulators in cell cycle progression during SAM development.

## Methods

### Plant materials and growth conditions

*ccs52a2-3*, *ccs52a2-1* and *ccs52a2-2* mutants (Col ecotype) in the *CCS52A2* gene were obtained from the SALK_074403, SALK_073708 and SALK_001978, respectively, from the Arabidopsis Biological Resource Center (ABRC). *wus-1*, *clv1-1*, *clv1-4*, *clv3-2*,CLV::GUS marker line, WUS::GUS marker line (all in L*er* ecotypes), *mgo1-1* (Wassilewskija, Ws ecotpye) and *fas1-1*(L*er* ecotype) were used. In the case of double mutants with *wus-1*, *clv1-1*, *clv1-4*, *clv3-2*, CLV::GUS marker line, and WUS::GUS marker line, we analyzed only plants that also harbored the *erecta* mutation. To ensure there were no background effects with the *ccs52a2-3* phenotype, we crossed ccs52a2-3 with wildtype Ler accession, and selected ccs52a2-3 erecta plants for examination. *ccs52a2-3* mutation showed no significant difference in phenotype between these two ecotypes. All of the plants for morphological analysis were grown in soil in growth chambers under 14-h-light/10-h-dark photoperiods (120μmolm^-2^ s^-1^) at 24/22 degrees C. Seedlings used for microscopic studies were grown on Murashige and Skoog (MS) medium on vertically oriented plates in growth chambers under the same light conditions. The F_1_ of all double mutants were morphologically similar to WT. Seeds from plants with the *ccs52a2-3* phenotype in the F_2_ population were chosen to harvest, and *clv*, *wus*, *fas1* or *mgo1* phenotypic plants were selected as homozygous double mutants in the F_3_ population. In F1 and F2 population of the double mutant, the *clv*, *wus* and *ccs52a2-3* mutation was also genotyped by sequencing, which were carried out with the following primers: DWUSL: 5’-CTACCACCGTTGATGTGATC-3’; DWUSR: 5’-TCATGCAAGCTCAGGTACTG-3’; DCLV1L: 5’-GACTTCTTGGTTACGTAGCG-3’; DCLV1R: 5’-ACATCACTCTTCTCGTCCAC-3’; *ccs52a2-3* mutation was genotype by checking the PCR product in agarose gelwith primers: 35R-2: 5’-GGTGATTAGTTACCCATCCACGTGTTATAC-3’; 38 F-2: 5’-GCACTTGCTAGCTTACTTCACCGAATCATA-3’.

### RT-PCR and Real-time PCR

RNA was extracted from various tissues using the Trizol method, and 2 ug of RNA was used with M-MLV reverse transcriptase (Promega) in an RT reaction to cDNA. The expression level of each gene was normalized against the expression level of ACTIN2. Reverse-transcript PCR was carried out with the following primers: CCS52A2R: 5’-TCGTAACACATCCCATATCT-3’; CCS52A2F:5’-TAAGACGGAAACGCAGAGGT-3’; and ACTINF: 5’-TGCGACAATGGAACTGGAATG’-3; ACTINR: 5’- CAAbGACGTAGGATAGCATGTG-3’. Primers for qRT PCR reactions: qWUSR/F, qCLV3R/F were synethesised by previously report [41]; qCCS52A2F: 5’-ACACGCCGCAGCAGTGA-3’; qCCS52A2R: 5’-AGCAGTGCCACCACCAGAAG-3’; qACTIN2F: 5’-GGCTCCTCTTAACCCAAAGGC-3’; qACTIN2R :5’-CACACCATCACCAGAATCCAGC-3’.

### Tissue sectioning and GUS staining

Tissue was fixed in FAA (3.7% formaldehyde: 50% ethanol: 5% acetic acid) overnight and embedded with Technovit 7100 (Heraeus Kulzer) following the protocol from manufacturer. The sections were cut to 2um and stained with 0.1% toluidine blue. The slides were washed with 70% and 100% ethanol and embedded with a coverslip by neutral resins after drying in air. Histochemical GUS was performed as described previously (Schoof et al., 2000) with minor modifications (elongated the staining time to 8–10 hours).

### *In Situ* Hybridization

The shoot apices of 8-day-old seedlings and 28-day-old plants were fixed for 3 h in FAA (3.7% formaldehyde: 50% ethanol: 5% acetic acid) solution after vacuum filtration and embedded in paraplast embedding medium (Oxford Labware, St. Louis, MO). Samples were sectioned at 8um thickness and placed onto Probe-On Plus microscope slides (Fisher Scientific). Tissue preparation, hybridization and detection procedures were performed as described [13,42]. All the probes were generated with the DIG RNA Labeling Kit T7/SP6 (Roche) and T3 RNA polymerase (Promega), following the manufacturer’s instructions. The template of the *CLV3* probe was the first 291 bp of the *CLV3* coding sequence and was cloned into the pCRBlunt vector (Invitrogen). The template of the *WUS* probe was the first 664 bp of the *WUS* coding sequence and was cloned into the pBluescript KS vector (Fermentas). The *CCS52A2* probe was generated by cloning the 340 bp of 5’ UTR sequence into pGEMTeasy (Promega), with the primers CCS525UL: 5’-CCCGGGAAAGAAAAAAAAAAC-3’ and CCS525UR: 5’-ACTCGAGTCACGAATCAA-3’. All templates for the probes were sequenced before the probes were synthesized.

### Construction of Transgenes

To generate the transgene for complementation, the full-length genomic sequence (9.7 Kb) of *CCS52A2*, including the *CCS52A2* coding region (including introns), plus a 1.5-Kb promoter and 1.2-Kb terminator, was excised from BAC T26M18 (provided by ABRC) with restriction enzymes XbaI and EcoRV and inserted into the XbaI-SmaI site of the pCambia 3301 vector. The plasmids were introduced into *Agrobacterium tumefaciens* strain GV3101 by electroporation and transformed into *ccs52a2-3* heterozygote plants by the floral dip method [43,44]. The positive transgenic *ccs52a2-3−/−* plants were screened from 12-day-old T_1_ generation by genotyping and spraying 54 mg/ml Basta.

To generate the CCS52A2_P_::CCS52A2_CDS_::GUS constructs, the 1.6 Kb promoter region of *CCS52A2* was amplified using primers PG1R: 5’-GTCAAGCTTTGTTAACCGTGAAGGCTCTG-3’ and PG1L: 5’-CCGTCTAGATTACTGTTTCGTTC CTCCAG-3’, digested with Hind III and XbaI and inserted into pBI121. Then a 1.5Kb CDS region was amplified from cDNA using primers p1ATGL2: 5’-GCGTCTAGATGGAAGAAGATGAATCAAC-3’ and p1ATGR2: 5’-TATGGATCCACACCG GATTGTTGTT CT-3’, digested with BamHI and Xbal and inserted into pBI121 after the promoter. All the plasmids were introduced into *Agrobacterium tumefaciens* strain GV3101 by electroporation and transformed into wild type (Col) or *ccs52a2-3* heterozygote by the floral dip method [43,44].

## Competing interests

The authors declare that they have no competing interests.

## Authors’ contributions

YJL carried out all of the experiments (except as noted below), constructed all of the figures, and wrote the manuscript. WY participated in molecular cloning and promoter-GUS analysis. BBL isolated the *xcm9* mutant and carried out preliminary mapping of the gene. XJZ participated in the screening the double mutant. KDL and MPR designed the experiments and supervised the work. All authors read and approved the final manuscript.
